# Surgeon-Directed Neuromonitoring in Adolescent Spinal Deformity Surgery Safely Assesses Neurological Function

**DOI:** 10.7759/cureus.19843

**Published:** 2021-11-23

**Authors:** Andrea Chan, Purnajyoti Banerjee, Cristina Lupu, Tim Bishop, Jason Bernard, Darren Lui

**Affiliations:** 1 Orthopaedics, St George's Hospital, London, GBR; 2 Trauma and Orthopaedics, Kettering General Hospital, Kettering, GBR; 3 Orthopaedics, St. George's Hospital, London, GBR

**Keywords:** spinal surgery, surgeon directed neuromonitoring, neuromonitoring, paediatric surgery, adolescent idiopathic scoliosis (ais)

## Abstract

Background

Spinal deformity correction is associated with the risk of intra-operative neurological injury. Surgeon-directed monitoring (SDM) of transcranial motor-evoked potentials (TcMEP) is an option to monitor intra-operative spinal cord function. We report a retrospective analysis of a prospective database to assess the safety of this technique in spinal deformity correction in adolescent patients.

Methods

Surgeon-directed neuro-monitoring was utilised in 142 consecutive deformity correction surgeries (2012-2017). Surgeons were responsible for electrode placement, intra-operative stimulation, and interpretation of TcMEP data. If waveform disappearance occurred in the lower limb (LL), the surgeon would re-stimulate after excluding technical or anaesthetic factors. Failure to return normal waveforms led to maneuver reversal and reducing distractive force and ensuring subsequent return to baseline. Wake up test and ankle clonus followed by staging surgery was considered if the LL waveforms failed to return indicating potential motor injury.

Results

Of 142 patients, three cases (2.11%) had a complete visual loss of LL signals that did not resolve with anaesthetic stabilisation, leading to reversed surgical manoeuvre and staged surgery. No cases with permanent neurological dysfunction were recorded. This outcome supports surgeon-directed monitoring as a safe monitoring option, as an alternative to neurophysiologist-led monitoring. It also provides evidence in support of the waveform disappearance criteria as a safe TcMEP warning criterion with a 100% negative predictive value.

Conclusions

Where there is a lack of availability of trained neurophysiologists, surgeon-directed neuro-monitoring is a safe and reliable method of preventing intra-operative neurological injury amongst adolescent patients undergoing deformity correction.

## Introduction

Background

Intraoperative neuromonitoring (IONM) provides surgeons with critical information regarding the neurological status of their patients during surgery. There are reports of iatrogenic intraoperative neurological damage in deformity correction ranging from 1.5% to 9% [1]. Neuromonitoring should alert surgeons to potential deficit outcomes in the anaesthetised patient, allowing for adjustment of anaesthetic, reversed surgical action, or other forms of intervention [2]. Monitoring of transcranial motor-evoked potentials (TcMEP) effectively serves this purpose [3] and is widely used for spinal cord monitoring in patients undergoing spinal deformity surgery. This method of TcMEP monitoring uses the patient’s upper limbs as controls and the lower limbs for spinal cord assessment.

Motor-evoked potentials are signals measured in the peripheries after high voltage, short-duration electrical stimulation of the motor cortex via electrodes placed on the patient’s scalp [2]. Motor cortex stimulation passes down the corticospinal tract to activate skeletal muscles, producing compound muscle action potentials (CMAPs). Muscle activation is measured by needle electrodes inserted at various locations on the upper and lower limbs [4]. These transcortical electrical stimulations offer real-time instant monitoring of the integrity of the entire motor pathway and detect potentially reversible neurodeficits through spinal cord ischaemia secondary to vascular compromise [5]. Drawbacks to TcMEP monitoring include the specific and rigid anesthetic protocols - neuromuscular blockade and halogenated anesthetic agents cannot be used. It is also not suitable for use in patients with epilepsy, cardiac pacemakers, cochlear implants, or raised cerebral pressures. Monitoring is discontinuous as stimulation is intermittent. A bite guard is also mandatory to avoid tongue biting due to cortical stimulation [5].

Other forms of monitoring include somatosensory evoked potentials (SSEPs), which examine the ascending dorsal sensory tracts. Peripheral nerve stimulation evokes cortical, subcortical, or brainstem response [2]. Subcortical recording sites are valuable in patients with cortical abnormalities. Limitations to SSEPs include delayed detection of cord injury, inability to localize neurological damage, sensitivities to anesthetics, and inability to detect damage to motor pathways.

EMG allows for specific monitoring of individual myotomes, and recordings can be spontaneous or triggered [2]. Similar to TcMEPs, ascending signals are recorded through subdermal needles placed in corresponding muscle groups. A stimulus is applied until the threshold necessary to trigger a CMAP is reached. If the cortex is breached during spine manipulation or instrumentation, nerve root irritation decreases the threshold to cause a CMAP. This method is less sensitive to inhaled anesthetic agents, but muscle relaxants must be avoided.

Rationale

A surgeon-directed version of TcMEP monitoring is used in our spinal deformity correction unit. Ideally, the persistence of a single parameter derived from TcMEP signals correlated with motor outcomes in patients. Monitoring this parameter would then give surgeons confidence to continue surgery [6]. Unfortunately, there lacks evidence for any single parameter to be used alone as a warning criterion [7]. There are three main paradigms: the disappearance of previously present TcMEP signals, TcMEP amplitude reduction, and increase in stimulus voltage threshold. The disappearance criterion can be described as a loss of visible TcMEP signal at unchanged display sensitivity, adjusted to maximise responses [6]. Signal disappearance is reportedly frequently pathological and a strong indicator for temporary or long-term paresis [7,8]. Amplitude reduction criteria warn against reduction in still-present TcMEP signals by >80% [2]. The theoretical explanation is that conducting capacity of corticospinal fibers and or recruitment of motor units may be diminished in pathology, hence amplitude deterioration indicates neurological injury [6]. The increasing threshold required to elicit response may also indicate pathology as fibers with the lowest activation threshold are highly susceptible to damage.

We reviewed the use of the TcMEP waveform disappearance criterion in consecutive cases of paediatric spinal deformity correction in our spine deformity unit from 2012-2017 undertaken by orthopaedic spine surgeons and explore its efficacy and safety. SSEPs and EMG monitoring were not used in conjunction to TcMEP monitoring. We also describe a surgeon-directed approach to TcMEP monitoring, in contrast to neurophysiologist-led monitoring. The use of this approach is becoming more widely as reflected by emerging literature over recent years.

## Materials and methods

A retrospective review was undertaken of consecutive paediatric spinal deformity correction surgeries between 2012 and 2017. A total of 142 cases were identified in which surgeon directed version of motor evoked potential monitoring system was utilized to monitor spinal cord integrity.

Intraoperative monitoring

After anaesthetization and prior to surgery, the surgeons applied the stimulating electrodes across the patient’s scalp (over motor cortex), and the receiving electrodes to the peripheral muscles in the anesthetized patient. Once the patient was positioned and prepped, this was followed by pre-procedure stimulation to establish a baseline signal pattern. At this point, display monitor settings may have been adjusted if necessary, to maximize signal response.

During surgery, the surgeon was responsible for intermittently pausing the procedure for stimulation and interpreting the signals shown on the screen of the monitoring device. Stimulation of transcranial TcMEPs were repeated at the surgeon’s discretion, particularly after maneuvers including insertion of hardware or distraction.

We used the relationship between upper and lower limb signal traces as our warning criteria [8]. A true event (TE) was defined as a complete visual loss of lower limb (LL) TcMEP trace without reduction in the ipsilateral upper limb (UL) TcMEP amplitude. This would suggest that the corticospinal tract inferior to the cord levels supplying the upper limb had been disrupted. The most likely cause of this was intraoperative maneuvers in the thoracolumbar region where corrective instruments were exerting traction on the spine and spinal cord. If both upper and lower limb TcMEP traces were lost, this was likely caused by nonsurgical anesthetic confounding factors.

**Figure 1:**
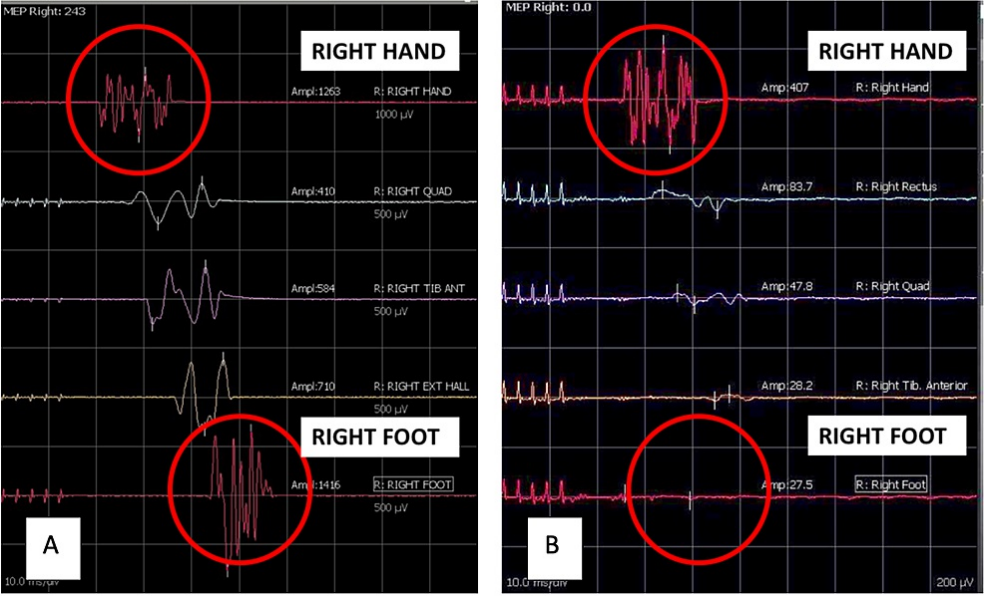
Stimulation data from right-side of body. Normal waveforms in all limbs (A). Example of true event ‘TE’: loss of LL signals with the persistence of UL signals (B). LL: lower limb; UL: upper limb.

Figure 1 shows an example of a TE, where UL and LL waveforms are both present in the first stimulation (Figure 1A), but LL waveform is lost in the second stimulation (Figure 1B).

The operating surgeon recognized an intraoperative TE (right, left or bilateral) as loss of LL signal amplitude on the monitoring screen, described as the disappearance criterion by MacDonald [6]. Following observation of a TE, measures including re-stimulation, adjustment of anesthetic, or reversed surgical action were taken in an attempt to normalize signals.

**Figure 2:**
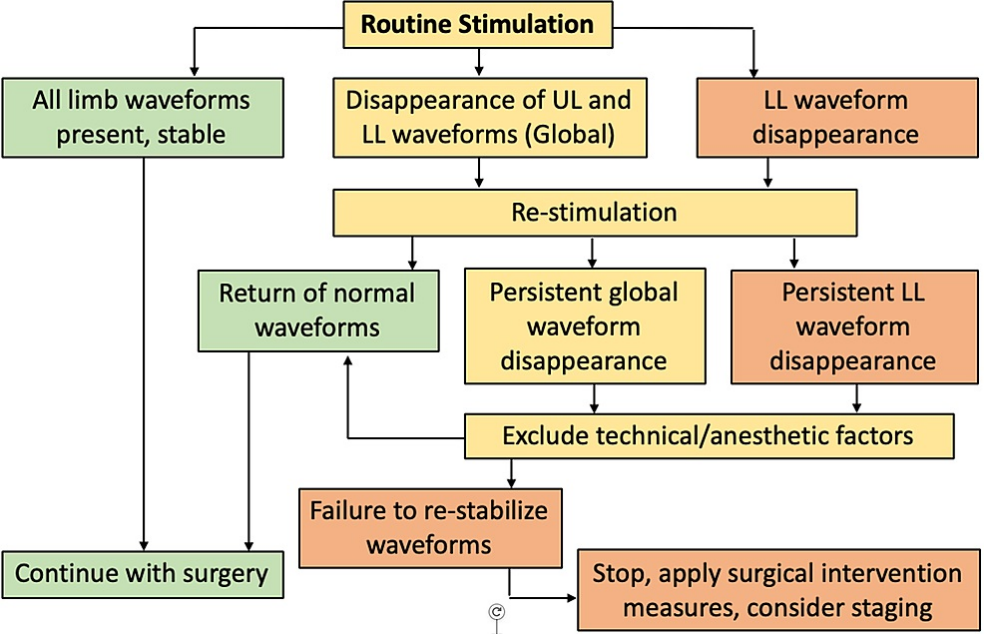
Monitoring method used at SGH. Outlines possible outcomes of routine stimulation during surgery, and course of action taken for each outcome. LL: lower limb; UL: upper limb.

The flowchart in Figure 2 describes the monitoring method used in our unit. The aim was to keep TcMEPs stable throughout surgery (green pathway). If waveform disappearance occurred, the surgeon would re-stimulate, then exclude technical/anesthetic factors. Technical-related factors included the electrodes and patient positioning, while anesthetic-related factors included hypotension, hypoxia and hypothermia.

If this failed to return normal waveforms, interventions involving maneuver reversal, reducing distraction, and restimulation were taken. A wake-up test and ankle clonus were performed if the waveform failed to return to baseline, followed by the staging of surgery.

Anesthetic technique

Total intravenous anaesthesia was used in all surgeries, due to the known effect of halogenated anesthetics on TcMEP monitoring. 

## Results

Patient baseline data is described in Table 1. Indication for surgery by type of spinal deformity is described in Table 2. The most common type of deformity operated on was adolescent idiopathic scoliosis, representing 85% of cases.

**Table 1:** Patient baseline characteristics.

Baseline characteristics	
Number of cases	142
Age, years (range)	13.9 (5-17)
Female patients, n	114
Male patients, n	28
Mean pre-op cobb angle, ° (SD)	61.1° (19.6°)

**Table 2:** Number of each type of spinal deformity. Percentages are approximated to integer numbers.

Classification of spinal deformity	
Adolescent idiopathic scoliosis (%)	120 (85%)
Syndromic scoliosis (%)	9 (6%)
Neuromuscular scoliosis (%)	9 (6%)
Scheuermann’s kyphosis (%)	2 (1%)
High-grade lumbar spondylolisthesis (%)	2 (1%)

Reliable signals were achieved at the beginning of surgery in all cases. Mean duration of surgery was 302.5 minutes (SD 105.7 minutes) with an average of 20 stimulations per case.

All corrections were achieved with multilevel polyaxial pedicle screw insertion and contoured rods in the thoracolumbar spine.

One hundred forty-two patients, including those who required staging, were without permanent neurological deficit post procedure. This demonstrated a negative predictive value of 100%.

There were three notable cases (2.11%) where TEs were observed with persistent disappearance of LL TcMEP signal despite initial re-stimulation and signal correction attempts.

1. Patient 1 had an intraoperative TE on the left side, leading to reversal of corrective maneuver. On waking, neurological examination confirmed weakness on the left side. Neurology recovered post-operatively. T4-T12 scoliosis correction was completed in follow-up procedure three days later. No neurological deficit was noted at post staged procedure.

2. Patient 2 had an intraoperative TE that was attributed to the penetration of the thoracic canal due to malposition pedicle screw on imaging. Surgery completion was staged three days later to allow for neurology to stabilize. No neurological deficit post staged procedure.

3. Patient 3 had an intraoperative TE caused by the placement of a pedicle screw causing compression of the canal. Surgery was staged twelve days later. No neurological deficit post staged procedure.

Average post-op angle percentage correction of 64.7% (SD 15.8%) without any significant spinal cord injury using the surgeon-controlled IOMN.

## Discussion

SDM of TcMEPs can deliver safe surgery in deformity correction surgery. Real-time monitoring of spinal cord integrity with 100% negative predictive value provides reassurance for the surgeon. Without a neurophysiologist present in the theatre, the surgeon assumed the responsibility for placing the electrodes, establishing signal baseline, and leading stimulation and interpretation of TcMEP signals. When the surgeon is experienced in using this method of monitoring, there is little disruption to the flow of surgery.

The warning criteria used to interpret TcMEP signals was the disappearance of the LL waveform with the maintenance of the UL waveform. LL signal disappearance was most commonly found after hardware insertion and cord distraction. In patients who did not tolerate cord manipulation and repeatedly demonstrated signal changes during initial surgery, staged surgery allowed for physiological recovery and subsequent surgery completion.

Expert review on the interpretation of TcMEPs by Kothbauer [9] concluded that the best correlation of TcMEP data to postoperative clinical deficit lies in assessing the disappearance of previously present TcMEPs, regardless of threshold or amplitudes. However, they also stress elevated threshold and amplitude reduction may be useful as minor warning criteria, indicating sub-clinical injury.

The absence of postoperative neurological deficits in the 142 patients in this study provides evidence to support the reliability of the disappearance criterion, in the context of LL TcMEP signal disappearance in deformity correction surgery. Positive patient outcomes imply that all TEs were identified, and a 100% negative predictive value.

A recent study by Magampa and Dunn [10] similarly found that using the literature suggested percentage amplitude changes as an indication of cord damage was less useful than comparing the amplitude of the hands and legs. Instead, close to total loss of leg signals with maintained hand signals was a reliable method with a favorable outcome.

Among the team, the anesthetists are especially valuable in working closely with the surgeons. Their knowledge and skill in delivering total intravenous anesthetic allows TcMEP monitoring to be used. They are also responsible for monitoring and maintaining patient temperature and blood pressure. Changes in these parameters are communicated to the team to prompt TcMEP stimulation.

The responsible person for overseeing neuromonitoring varies between centers, and may include specialist neurophysiologists, neuro-anesthetists [11], or spinal surgeons. Although the use of intraoperative neuromonitoring has become more widespread and increasing literature has emerged describing its safety [5], the biggest limitation appears to be the shortage of skilled staff who can employ and interpret monitoring signals [5,11,12]. Ray [5] describes adopting the ‘Surgeon directed TcMEP mode’ as a viable alternative. This approach has also been supported in other published case series by authors including Biscevic et al, and Magampa and Dunn [10,12].

The Scoliosis Research Society statement on neuromonitoring [13] concluded that the use of intraoperative spinal cord monitoring during corrective surgery is considered optimal care, and its adoption is strongly recommended. They also state that IONM should be performed by practitioners who are skilled at the technical and interpretative aspects of monitoring to allow real-time response to changes in signals. SRS endorses the use of a standardized protocol or checklist when an intraoperative alert occurs. The protocol used at this trust was described in Figure 2 in our method. There are many similarities to the consensus statement developed by Vitale et al [14] and SRS supports it. 

While there are a number of board certifications for IONM in the United States, there is no standardized certification for accepted practice in other countries. There is currently a need for standardized certification for IONM monitoring in other countries. 

A major limitation of this study was that the incidence of visual signal loss and surgical intervention was not recorded prospectively. Therefore, rate of false-positive results or positive predictive value of signal loss warnings cannot be reported. 

Signal data revealed 59 other patients also experienced LL TcMEP amplitude reductions of >95%; they experienced amplitude reductions equal to or greater than the three known deficit cases. The operating surgeon would have observed the same disappearance of LL waveforms on the computer screen in these cases and intervened accordingly to avert injury. It may be suggested that these patients also had TEs (27 right, 26 left, 15 bilateral) but were able to recover LL signals by successful intervention measures. However, we can only speculate which warnings were due to reversible surgical causes, and which warnings were false positives due to confounding factors e.g. anesthesia, hypotension, hypothermia, mispositioning or metabolic disturbances.

Prospectively recording TcMEP warnings incidence under the three descriptions below would remove speculation and produce more convincing evidence on the specificity and positive predictive value of warnings at a given amplitude reduction level. 

(1) Temporary loss of signal due to nonsurgical causes - false positive

(2) Temporary loss of signal due to surgical causes - true positive (corrected intraoperatively)

(3) Persistent loss of signal - true positive (requiring staging).

This study was able to identify patients in group three, but unable to make the important distinction between groups one and two which would have contributed to evaluation of criterion performance.

Finally, the outcomes described in this study heavily depended on the experience and confidence of the surgeons. The spinal surgeons in our unit have been using this technique of monitoring neurophysiology for many years. They have built their skill and experience in using this monitoring approach.

## Conclusions

This study has shown that SDM, using the disappearance criterion is a safe method of TcMEP monitoring. In addition, this method has the potential to be a reliable alternative to traditional neurophysiologist-directed neuromonitoring for children undergoing deformity correction surgery. We have used this technique to undertake surgeon-controlled neuromonitoring in patients with incomplete spinal cord injuries secondary to trauma and tumours or in cases of compression from stenosis such as cervical myelopathy. This enhances the team's ability to monitor spinal cord function during surgery in the acute setting and facilitates appropriate intervention. Furthermore, this service can be delivered outside regular working hours in an on-call setting without reliance on out-of-hours neurophysiology. However, we recognise that not all deformity correction cases are equal in terms of patient complexity and the experience of the surgical team. Patient safety should always be prioritised, and in more complex cases, operating surgeons should use their judgment to decide whether to request a neurophysiologist's expertise.
